# High-Intensity Interval Resistance Training (HIRT) influences resting energy expenditure and respiratory ratio in non-dieting individuals

**DOI:** 10.1186/1479-5876-10-237

**Published:** 2012-11-24

**Authors:** Antonio Paoli, Tatiana Moro, Giuseppe Marcolin, Marco Neri, Antonino Bianco, Antonio Palma, Keith Grimaldi

**Affiliations:** 1 Department of Biomedical Sciences, Physiological Laboratory, University of Padova, via Marzolo 3, Padova 35131, Italy; 2 Italian Fitness Federation, Ravenna, Italy; 3 Department of Sports and Exercise Science (DISMOT), University of Palermo, Palermo, Italy; 4 Biomedical Engineering Laboratory, Institute of Communication and Computer Systems, National Technical University of Athens, Athens, Greece

**Keywords:** Resistance training, Resting energy expenditure, Interval training, Respiratory ratio

## Abstract

**Background:**

The benefits of exercise are well established but one major barrier for many is time. It has been proposed that short period resistance training (RT) could play a role in weight control by increasing resting energy expenditure (REE) but the effects of different kinds of RT has not been widely reported.

**Methods:**

We tested the acute effects of high-intensity interval resistance training (HIRT) vs. traditional resistance training (TT) on REE and respiratory ratio (RR) at 22 hours post-exercise. In two separate sessions, seventeen trained males carried out HIRT and TT protocols. The HIRT technique consists of: 6 repetitions, 20 seconds rest, 2/3 repetitions, 20 secs rest, 2/3 repetitions with 2^′^30″ rest between sets, three exercises for a total of 7 sets. TT consisted of eight exercises of 4 sets of 8–12 repetitions with one/two minutes rest with a total amount of 32 sets. We measured basal REE and RR (TT_0_ and HIRT_0_) and 22 hours after the training session (TT_22_ and HIRT_22_).

**Results:**

HIRT showed a greater significant increase (p < 0.001) in REE at 22 hours compared to TT (HIRT_22_ 2362 ± 118 Kcal/d vs TT_22_ 1999 ± 88 Kcal/d). RR at HIRT_22_ was significantly lower (0.798 ± 0.010) compared to both HIRT_0_ (0.827 ± 0.006) and TT_22_ (0.822 ± 0.008).

**Conclusions:**

Our data suggest that shorter HIRT sessions may increase REE after exercise to a greater extent than TT and may reduce RR hence improving fat oxidation. The shorter exercise time commitment may help to reduce one major barrier to exercise.

## Background

Daily energy expenditure may be divided into different components that can be categorized as a) resting metabolism, b) thermic effects of food and c) the energy expenditure of physical activity associated with exercise and non-exercise movement [1,2]. An analysis of the literature shows that in Western countries the mean ratio of daily energy expenditure and resting energy expenditure (REE) is 1.66; this means that only 40% of energy is expended on activity while the remaining 60% is expended at rest [3]. It has been calculated that three 30 min sessions of vigorous exercise per week increase energy demands by 1,039 Kcal/week (i.e. only 5.3% of the average weekly expenditure 19,562 Kcal/week) [3] but to achieve a level of energy expenditure comparable with that of humans living in primitive societies it would be necessary to exercise at high intensity for 90 minutes every day. It is evident that this amount of time cannot be proposed to the average population, even exercising 30 minutes per day, three times a week involves a high rate of drop out [4]. Notwithstanding these considerations, clinical practice and data from the literature consistently point to the beneficial effect of even just some exercise on fat loss and other health aspects [5]. The only way to reconcile the apparently contradictory evidence between experimental and clinical data is to hypothesize that other exercise related factors are involved in the fat-loss effect other than the simple increase in energy expenditure during exercise. Hence we should consider basically three fundamental mechanisms that may play a role in this complex interrelationship, a) exercise might reduce hunger, b) exercise might improve fitness levels and consequently might change behaviour related to non-exercise activity thermogenesis such as walking, stair climbing, etc. [2,5], c) a positive effect of exercise on resting metabolism - the latter is the subject of our study. As underlined above resting energy expenditure (REE) is the largest component of the daily energy budget and, consequently, any increase in REE in response to exercise could potentially have a great impact on health promotion and weight control. In recently published weight control guidelines resistance training (RT) has been incorporated as an important component of exercise protocols [6,7]. RT acts in a substantially different way compared to endurance training (ET), it increases muscle mass in the long term [8] but also increases excess post-exercise oxygen consumption (EPOC) immediately after the training session [9]. As Gaesser and Brooks identified in 1984 oxygen consumption (VO_2_) decreases exponentially following an exercise session, starting from this observation they defined the recovery period in which an increase in oxygen uptake is observed as “excess post-exercise oxygen consumption” (EPOC) [10]. This elevated post-exercise metabolism plays a part in the energy cost of exercise and influences the thermic effect of activity. Numerous studies have demonstrated the effect of endurance training on basal metabolic rate and on respiratory ratio, but data from research on resistance training and EPOC are conflicting. The difficulty in measuring energy expenditure during non-steady-state intermittent physical activity may be one of the reasons for this lack of a substantial body of literature, but the main point is that several different types of RT training protocols are confounded under the umbrella name of “resistance exercise” (e.g. circuit training or multiple sets), so it is difficult to quantify how the particular type of weights, sets, repetitions, and length of rest periods utilised [11,12], influence the energy cost of the exercise [13]. Recently increased interest has been shown in the concept of HIT (High-Intensity Interval Training) investigated by Gibala and his group [14]. Low-volume HIT is characterized by brief repeated ‘bursts’ of vigorous exercise interspersed with periods of rest or low-intensity exercise for recovery. It is notable from these studies and the evidence that demonstrates the close connection between EPOC and exercise intensity that there are surprisingly few reports comparing the different kinds of strength training techniques. The aim of our study was to investigate whether and how two different kinds of resistance training, a traditional and a high-intensity resistance protocol, affect resting energy expenditure and respiratory ratio 22 hours after the training session.

## Methods

### Study participants

18 resistance-trained males (28 ± 4.5 yrs old, 4–6 yrs training experienced), height 174 cm (±3), weight 84 Kg (±3) responded to an invitation to participate in the study (Table 1). Respondents provided written informed consent and were screened for the presence of disease or conditions that could place them at risk of an adverse response to exercise. Subjects were not taking any medications and had medium experience in resistance training (±3.5 years) so familiarization sessions were not necessary. Muscle and fat quantities & percentages were assessed by skin fold measurements which are highly correlated with percent body fat in fit and healthy young men [15]. We used software (Fitnext®, Caldogno, Vicenza, Italy) that includes 9 skinfolds (triceps, biceps, pectoral, subarmpit, subscapular, iliac crest, mid-abdominal, anterior thigh, medial calf), 6 bone circumferences (arm, forearm, waist, hip, thigh, calf), 4 bone diameters (elbow, wrist, knee, ankle), waistline and hip circumference measurements [8]. Anthropometric measurements were performed according to the Anthropometric Standardization Reference Manual [16]. Weight was measured to the nearest 0.1 kg using an electronic scale (Tanita BWB-800 Medical Scales, USA), and height to the nearest 1 cm using a Harpenden portable stadiometer (Holtain Ltd, UK). Skinfolds were measured to the nearest 1 mm using a Holtain caliper, and circumferences to the nearest 1 mm using an anthropometric tape. All measurements were taken by the same operator (LC) before and during the study according to standard procedures [16]. One subject withdrew from the study for personal reasons. The study was approved by the Ethical Board of the University of Padova Department of Biomedical Sciences and conformed to standards for the use of human subjects in research as outlined in the current Declaration of Helsinki. Investigators explained the purpose of the study, the protocol to be followed, and the experimental procedures to be used prior to allowing participants to enter the study.

**Table 1:** Physical and anthropometrical characteristics of the subjects

**Variable**	**mean**	**SD**
Age (years)	28	4.5
Height (cm)	174	3
Body mass (Kg)	84	3
Body fat (%)	8.5	4.7
Muscle (Kg)	51	6.2

### Study design

The 17 subjects performed a 6-RM test with the various exercises included in the training schedule as described elsewhere [17]. A 6 RM test is suitable for testing maximal strength in subjects with little or no previous resistance training experience. Data obtained from the initial test was used to determine an appropriate starting level for resistance training. This technique has been shown to have high reproducibility (r = 0.99). Subsequently subjects did a specific warm-up for each 1RM test by performing 5 repetitions with a weight they could normally lift 10 times. Using procedures described elsewhere [18] the weight was gradually increased until failure occurred in each of the exercises tested for rest pause (leg press, bench press, traction at dorsal machine). The higher load was considered the 1 RM [18]. The test-retest reliability in our laboratory for 1RM varies from 0.92 to 0.97 (ICC). A 10 minutes warm up (treadmill run at 10 Km/h) was performed prior to training. The following week exercise trials were carried out to determine the appropriate load to complete 6 repetitions in the HIRT exercise. Using a trial and error modified methodology [18] starting with 40% and then after 5 minutes rest increasing the resistance by 5%. All subjects were able to complete 6 repetitions at 80–85% of the 1RM. The same methodology was used to assess the load that enabled the subjects to perform 12 repetitions (65–70% 1RM). The HIRT technique consisted of three series of 6RM followed by rest for 20 seconds; then the subject lifts the same weight until reaching the point of failure (habitually 2 repetitions) followed by 20 seconds rest then another 2/3 repetitions [8,19-21]. This sequence counted as one set, then subjects rested 2^′^30″ before performing a second and third set. Leg exercise included 3 sets with 2^′^30″ of rest between sets [21,22] whilst pectoral and latissimus dorsi consisted of two sets. The training session lasted approximately 32 minutes (including the warm up period).

In the TT session, using a modified exercise protocol [23], subjects performed four sets of eight different weight-lifting exercises and the intensity of each lift was set between 70% and 75% of their pre-established 1-RM. Subjects were instructed to perform as many repetitions as possible in a set, the usual number of lifts before failure was between 8 and 12 with one minute of rest between sets for single-joints exercises and two minutes for multiple-joint exercises. The protocol included: bench press, dorsal machine, military press, bicep curls and triceps extensions, leg press and leg curls, and sit-ups [6]. The training session lasted approximately 62 minutes (including the warm up period). The cadence between repetitions during both protocols was controlled [19]. As expected the muscle action velocity varied between subjects due to their different anatomical leverage, also there was a slight difference between repetitions for the same subject. However the average time of movement was superimposable and it was calculated as approximately 1.0 seconds for the concentric phase and 2.0 seconds for the eccentric phase.

**Figure 1:**
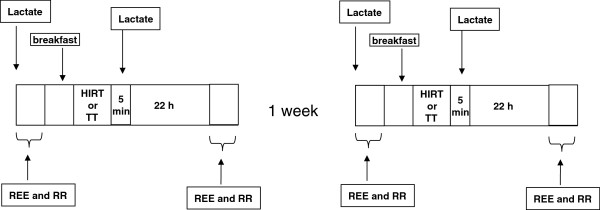
Scheme of experimental design.

On the first day measurements were taken of anthropometrics, basal REE and RR as described below. After a standard breakfast (described elsewhere, see Paoli et al. 2011 [24], they randomly performed the traditional training (TT_0_) or the high-intensity interval training (HIRT_0_); immediately after which blood lactate was measured. After 22 hours the subjects were recalled in the laboratory to repeat the basal condition measurements of REE and RR (HIRT_22_ or TT_22_.) The study was a cross-over design and one week later, under similar conditions, the subjects that performed TT in the first session carried out HIRT in the second, and *vice versa.* During the three days before each training session and during the day after, participants were provided a standard diet that provided approximately 20% fat, 55% CHO and 25% protein. The diet was designed to meet the free-living energy requirement estimate (REE x 1.4-1.8 according to self-reported physical activity levels) [25] (Figure 1).

### Measurements

Resting energy expenditure (REE) was analyzed using oxygen uptake (V’O_2_), carbon dioxide production (V’CO_2_) and respiratory ratio (RR) measurements with an Ergocard® ergospirometer (Pacific Medical Systems, Hong Kong , S.A.R) and a ‘pitot tube’ pneumotacograph equipped with standard gas analysers [24]. The gas analysis was performed in the morning before breakfast (7–8 am), while the subjects were seated. The room was dimly lit, quiet and approximately 24 C. Oxygen uptake was measured (ml/min) and also normalized to body weight (ml/kg/min) and the respiratory exchange ratio (RR) was determined. After resting for 15 minutes, data was collected for 30 min and only the last 20 minutes were used to calculate the respiratory gas parameters [26]. The system was calibrated before each measure using calibration syringes and precision oxygen and carbon dioxide gas mixtures. Subjects were requested to abstain from caffeine or alcohol consumption for 24 h prior to the measurement. V’O2 data were converted to REE expressed in Kcal/d using appropriate RR values and established tables based on the Weir equation [27].

Blood Lactate was measured using SensLab Lactate Scout – Test strips (Bautzner Staβe 67; Leipzig, Germany) based on the capillary blood lactate [18] oxidised by redox reaction via electrode mediation. Blood samples were taken from earlobe after 5 and 10 minutes after the end of training sessions to measure the lactate peak [28]. To verify that none of the subjects modified their nutritional behaviour during the intervention protocol (the day before and during the test day) an assessment of dietary intake was performed and analysed using DietComp® (Caldogno, Vicenza, Italy) software demonstrating a substantial similarity [24].

### Statistical analysis

Data is expressed as mean and standard deviation. Bland-Altman plots and comparison of the test-retest measurements performed in our laboratory confirmed good reproducibility of the measurements for RR and V’O_2_ (ICC >0.85 and >0.9 respectively with p < 0.05). The sequence of the training sessions (HIRT or TT) was generated by a random function. Data analysis was performed using the software package GraphPad Prism version 4.00 for Windows, GraphPad Software, San Diego California USA. An ANOVA repeated measurements was conducted since in this kind of experimental design subjects serve as their own control and assuming four different time points [29]. The substantial overlapping of basal condition was checked by a paired t-test to exclude a carryover effect. Whenever significant differences in values occurred, a Bonferroni test post-hoc was used. P-values was set at 0.05.

## Results

**Figure 2:**
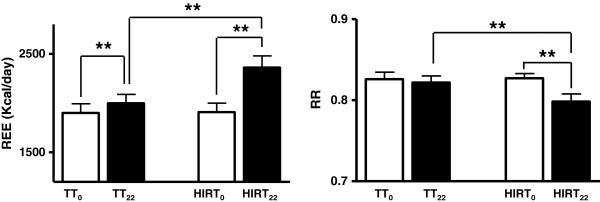
Resting energy expenditure REE and respiratory ratio RR before and 22 after high-intensity interval training and traditional training. ** = p < 0.001.

The total volume of work performed in the resistance training (loads x sets x repetitions) was significantly lower (P < 0.001) during the HIRT (3872.4 ± 624 Kg ) compared to the TT (7835.2 ±1013 kg). The mean level of maximal post-exercise blood lactate (Table 2) after HIRT (10.5 ± 2.1 mmol·L^-1^) was significantly greater (p < 0.05) than after TT (5.1 ± 1.2 mmol L^-1^). Table 3 describes Resting Energy Expenditure and Respiratory Ratio data during the recovery and 22 hours after the two training exercise interventions. No significant differences were measured between TT_0_ and HIRT_0_. As showed in Figure 2 there was a significant difference in energy expenditure for the TT exercise protocol (TT_0_1901 ± 93 Kcal/d vs TT_22_ 1999 ± 88 Kcal/d), and for HIRT where the difference even more marked (HIRT_0_ 1910 ± 89 Kcal/d vs HIRT_22_ 2362 ± 118 Kcal/d; p < 0.001). The comparison between TT22 and HIRT22 also demonstrated a significant difference (p < 0.001). At basal conditions, the values of RR in the two experimental conditions were similar (TT_0_ 0.826 ± 0.009; HIRT_0_ 0.827 ± 0.006) whilst a significant difference (P < 0.001) was found 22 hours after the training session in HIRT_22_ (0.798 ± 0.010) compared to both HIRT_0_ (0.827 ± 0.006) and TT_22_ (0.822 ± 0.008).

**Table 2:** La (blood lactate) mean values and SD before and after training

	**TT**_**before**_	**TT**_**afer**_	***P value***	**HIRT**_**before**_	**HIRT**_**after**_	***P value***	***P value TT***_***after***_***vs HIRT***_***after***_
**La (mmol/L)**	0.8 ± 0.2	5.1 ± 1.2	*<0.001*	0.8 ± 0.2	10.5 ± 2.1	*<0.001*	*<0.001*

P values of Bonferroni post hoc test are reported for before and after training sessions.

**Table 3:** REE (resting energy expenditure) and RR (respiratory ratio) mean values and SD at baseline and 22 hours after training sessions

	**TT**_**0**_	**TT**_**22**_	***P value***	**HIRT**_**0**_	**HIRT**_**22**_	***P value***	***P value TT***_***22***_***Vs HIRT***_***22***_
**REE (Kcal/d)**	1901 ± 93	1999 ± 89	*<0.001*	1910 ± 90	2362 ± 118	*<0.001*	*<0.001*
**RR**	0.826 ± 0.009	0.822 ± 0.008	*n.s.*	0.827 ± 0.006	0.798 ± 0.010	*<0.001*	*<0.001*

P values of Bonferroni post hoc test are reported for within and between training sessions.

## Discussion

To the best of our knowledge this is the first study to compare the effects of acute high-intensity interval resistance training with a traditional widely used training routine. The most interesting finding in this investigation was that V’O2 following HIRT remained significantly elevated even at 22 h post exercise and that this elevation was greater than has been reported before for other training protocols. Our results are in line with those of Schuenke et al. [9] except that in our case we observed a higher post exercise elevation of REE. Although the beneficial effect on health and weight control of a 24 hour increase in metabolism is evident [30] the optimal amount and type of exercise routine remains to be established. We recently proposed that resistance training should be investigated more thoroughly and rigorously by taking into account the variables involved including 1) muscle action used, 2) type of resistance used, 3) volume (total number of sets and repetitions), 4) exercises selected and workout structure (e.g. the number of muscle groups trained), 5) the sequence of exercise performance, 6) rest intervals between sets, 7) repetition velocity and 8) training frequency [11,12,21]. In the present study we chose a specific method of resistance training in order to simplify the interpretation and clarify the effect of RT on post exercise metabolism [11,12]. In recent years the HIT (high-intensity interval training) methodology has been widely studied by Gibala’s group [14] but HIT is based on a substantially cyclic, endurance type movement (e.g. cycling). Most commonly the sprints are performed on a stationary cycle ergometer at an intensity approaching 90% of maximal oxygen uptake (V^.^O2max). The most common protocol in published research is the Wingate test which consists of 30s of an all-out hard resistance sprint and subjects typically perform the Wingate test 4 to 6 times separated by 4 min of rest [14]. There are several reports that weight training may require more energy and a longer duration for recovery [31,32] compared to endurance training but despite this there are surprisingly few studies published on the specific influence of high intensity resistance training on metabolism. There are many factors that influence EPOC including for example the exercise order [33] but in particular the intensity and duration of exercise appears to be of greater importance. As Knuttgen asserted, the EPOC increased exponentially as a function of exercise intensity, whereas it increased linearly as a function of exercise duration [34]. Other studies reported that higher intensity resistance exercise generates greater EPOC than lower intensity resistance exercise [35,36]. Many authors [9,13,37] explain the basis of the greater increase in EPOC after more intense exercise as involving a perturbation of energy homeostasis. This consideration could help to explain the conflicting data between our study plus others reporting similar REE increases of between 16-20% [9,38,39] and several that reported a more modest increases (4–10%) [23,40-42]. Schuenke demonstrated an increase of 21.2% in 24 hours metabolism after a resistance training using a 8–12 repetitions per set with 2 minutes of rest between exercises [9] in circuits whilst a recent paper by Heden [41] has documented that one set or three set whole body training has the same effect on REE at 24 h post training (about 5%). This study used the ACSM guidelines: 10 repetitions, 10 exercises, divided into three circuit rotations each consisting of three or four different exercises with 30 sec of rest between each exercise. This kind of circuit training imposes by necessity a low total intensity of exercise. Our training protocol, on the contrary was performed at very high intensity [8,21] which can be demonstrated by the greater increase in maximal blood lactate levels and it is well known that lactate plays a role in the total increase of post exercise energy expenditure [43]. The greater level of lactate during the recovery phase in HIRT is evidence of a major metabolic stress derived from high intensity resistance training and may reflect the utilization of lactate as fuel in the aerobic pathway. But lactate removal may only be part of the process, in fact if lactate is infused during the post-exercise period it does not elicit a further increase in EPOC [44]. Lactate may explain, together with an increase in body temperature and the triacylglycerol cycle, only the short term component of EPOC [10,45]. Of the two phases in which EPOC can be divided: short term and long term, only the latter can explain the increase of resting energy expenditure registered 20–24 hours after training [46].

It is noteworthy that in our study, HIRT22 as well as registering a significantly higher REE also showed a lower RR. Regarding the latter Bahr et al. [45] taking into account the total energy expenditure and the rate of fatty acid oxidation from measurements of O_2_ uptake, respiratory exchange ratio, and urinary nitrogen excretion and the observation of a prolongation of the EPOC beyond one hour, claimed that triacylglycerol/fatty acid cycling is an important supporter of the energy cost in the prolonged component of EPOC because it is an indicator that the organism is using fatty acids rather than glycogen to satisfy the energy cost of exercise. Poehlman and Melby [47] added that the elevated fat oxidation noted during the recovery from any kind of resistance training seems to be a compensatory sparing of glycogen. Hence the significant lowering of RR after 22 hour after HIRT reflects an increased lipolytic effect. The Respiratory Ratio is a good way to identify the origin of energy substrates: when RR is close to 0.7 it means that the major energy source is lipids while when the ratio is near 1 carbohydrates are the main source of energy; in consideration of this, the results of our study, including a significant decrease of RR (from 0.827 ± 0.006 to 0.798 ± 0.010) 22 hours after HIRT whereas TT appeared to remain substantially unaltered, suggests that the HIRT might improve lipid metabolism at rest. The cause of this RR lowering could be explained in several ways: the more classical, cited above, is that glucose metabolic pathways are directed to replenish glycogen stores first [48] instead of being used for energy supply. This means that glucose and all gluconeogenic precursors will be spared from further oxidation and will be converted to glucose and glycogen, so lipids become the preferred oxidation substrate; the higher intensity of HIRT compared to TT is logically expected to produce a greater decrease of muscle glycogen. One intriguing hypothesis involves the AMPK/ACC (AMP kinases/ Acetyl CoA Carboxylase) relationship. It has been demonstrated that intense exercise [49] increases AMPK, thus AMPK can phosphorylate ACC decreasing its activity; the decreased ACC activity leads to a decrease in the rate of the synthesis of MalonylCoA and consequently there is a release of inhibition of CPT1 (Carnitine palmitoyltransferase I) activity leading to an increase in lipid oxidation [50]. Also the increase of ANP (atrial natriuretic peptide) stimulated by exercise could play a role in the increased rate of lipid oxidation; production of ANP is related to the intensity of exercise [51,52] and it has been demonstrated that ANP increases lipolysis [53], this pathway appears to be more suitable than the increase in catecholamines that have a very short half-life and appears not to be related to lipolysis after RT [54]. Growth hormone increase also could explain a part of the increase in lipid oxidation; it was demonstrated by Bottaro et al. [55] that intense exercise with incomplete recovery might stimulate GH production in significant manner. An exciting new hypothesis suggests that some cytokines and other peptides (myokines) that are produced and released by muscle fibres can exert autocrine, paracrine or endocrine effects that might influence the metabolic effect of exercise [56]. A recent paper describes a new polypeptide hormone, irisin, which is regulated by PGC1-α, it is secreted from muscle into the bloodstream and may activate thermogenic mechanisms in adipose tissue - this might also play a role in short time reported to lower RR [57].

As stated before reported REE after resistance training varies from 5-10% to 20%. These inconsistent results can be attributed to the different intensity and technique of RT used in the various studies. Our investigation showed an increase in basal metabolism after resistance exercise which we were also able to detect 22 hours after the training session. The increase of REE after TT was about 5% whilst after HIRT it was 23% and these data suggest an important effect on metabolism corresponding to 452 Kcal per day. Our data are consistent with those of Shuenke [9] and Melanson [38,39] but with higher values compared to previous studies. This difference may be explained by the higher intensity of our protocol although the physiological basis underlying this long lasting effect is still not completely understood. The effect on REE of an increase of muscle mass can be assessed only with longer training periods and not after a single bout of exercise. Some reports suggests that the β–adrenergic system may be involved in such an increase [42], while another explanation could be hormonal variations [9]. In fact, in response to exercise-induced trauma an increase of metabolic hormonal concentration is seen (e.g., cortisol, catecholamines, and thyroid hormone) that could increase metabolism. More likely increased protein re-synthesis due to post-exercise muscle damage is energy expensive (approximately 20% increase in resting metabolism) [58] and could contribute to greater EPOC after high intensity resistance training [43,59]. It could be speculated that the eccentric component of movement might have a much greater influence on the protein re-synthesis expense compared to the concentric movement but since in our protocol both exercise executions were superimposable regarding eccentric/concentric contractions, this aspect should be investigated further. The present study has a number of strengths. One is the novelty of our approach: the comparison between traditional and high intensity resistance training - to our knowledge this is the first report about the metabolic effects of High Intensity resistance Training. Our data demonstrate that one training session of HIRT appeared to have a more positive effect on metabolism during the day following the training session as the elevation of resting energy expenditure was maintained 22 hours after HIRT and at a higher level than for the TT session. The 22 hour Respiratory Ratio was lower in HIRT with respect to TT, reflecting an increased lipid oxidation at rest. Another strength of this study is that we propose a suitable protocol, with a small time commitment and significantly low volume, producing important metabolic and cardiovascular adaptations that could be a positive aid for weight control and fat loss and that may be useful in overweight subjects. HIRT is an atypical RT protocol: in HIRT subjects use heavy loads that induce mechanical effects on muscle but also use very short recovery periods comparable to an high intensity endurance training (like Hit). The main limitations of this study is that our subjects were mediumly trained and (as Laforgia notes *“The utility of supramaximal interval training for weight loss is nevertheless limited because this type of training is beyond the capabilities of non-athletes”*[13] could raise some concerns about the transfer of the methodology and results to sedentary/overweight subjects. In this regard, we and others [8,60], have demonstrated that it is possible, after a familiarization period, for previously untrained persons to successfully perform this kind of training – however it would be correct to exert appropriate caution when applying this protocol to overweight/obese subjects. Our next steps will be to verify the suitability of HIRT on a larger overweight/obese [8].

## Conclusions

Our results suggest that high-intensity interval resistance training increases excess post exercise energy consumption to a significantly greater extent than traditional resistance training. This exercise methodology allows subjects to improve metabolism and, at the same time, muscle mass and strength all of which are promoted as beneficial by many guidelines. In Western society leisure time is lacking and motivation to perform daily exercise is uncommon resulting in low overall levels of daily lifestyle related physical activity. In this situation a short intense training that enables elevation of basal metabolism whilst lowering RR (i.e. increase fat consumption at rest) may be an interesting and attractive alternative to more traditional and time consuming exercise and could be a useful tool in the physician’s hand. While the results of this study are encouraging further investigation is needed to explain the molecular pathways involved in such responses and the hormonal adaptation to HIRT.

## Abbreviations

HIRT: High-intensity interval resistance training; TT: Traditional training; REE: Resting energy expenditure; RR: Respiratory ratio; EPOC: Excess post-exercise oxygen consumption.

## Competing interests

Authors declare no competing interests

## Authors’ contributions

AP was the main researcher and was responsible for study design, statistical analysis and interpretation of data and draft of manuscript, conceived the study, participated in its design, drafted the manuscript and performed the statistical analysis. TM was responsible for study design and acquisition of data, GM was responsible for acquisition of data and participated in the statistical analysis, MN conceived the study and participated in its design, AB conceived the study and participated in its design, AP helped to draft the manuscript, KG participated in design of the study and helped to draft the manuscript. All authors read and approved the final manuscript.

## Funding

This study was supported by laboratory research funds at University of Padova.
